# Tannic Acid as a Bio-Based Modifier of Epoxy/Anhydride Thermosets

**DOI:** 10.3390/polym8090314

**Published:** 2016-08-26

**Authors:** Xiaoma Fei, Fangqiao Zhao, Wei Wei, Jing Luo, Mingqing Chen, Xiaoya Liu

**Affiliations:** The Key Laboratory of Food Colloids and Biotechnology, Ministry of Education, School of Chemical and Material Engineering, Jiangnan University, Wuxi 214122, China; feixiaoma24@163.com (X.F.); zhaofangqiao@gmail.com (F.Z.); wwei1985@jiangnan.edu.cn (W.W); jingluo19801007@126.com (J.L.); lxy@jiangnan.edu.cn (X.L.)

**Keywords:** tannic acid, epoxy resin, bio-based, toughening

## Abstract

Toughening an epoxy resin by bio-based modifiers without trade-offs in its modulus, mechanical strength, and other properties is still a big challenge. This paper presents an approach to modify epoxy resin with tannic acid (TA) as a bio-based feedstock. Carboxylic acid-modified tannic acid (TA–COOH) was first prepared through a simple esterification between TA and methylhexahydrophthalic anhydride, and then used as a modifier for the epoxy/anhydride curing system. Owing to the chemical modification, TA–COOH could easily disperse in epoxy resin and showed adequate interface interaction between TA–COOH and epoxy matrix, in avoid of phase separation. The use of TA–COOH in different proportions as modifier of epoxy/anhydride thermosets was studied. The results showed that TA–COOH could significantly improve the toughness with a great increase in impact strength under a low loading amount. Moreover, the addition of TA–COOH also simultaneously improved the tensile strength, elongation at break and glass transition temperature. The toughening and reinforcing mechanism was studied by scanning electron microscopy (SEM), dynamic mechanical analysis (DMA) and thermal mechanical analysis (TMA), which should be owned to the synergistic effect of good interface interaction, aromatic structure, decreasing of cross linking density and increasing of free volume. This approach allows us to utilize the renewable tannic acid as an effective modifier for epoxy resin with good mechanical and thermal properties.

## 1. Introduction

Because of their excellent performance properties, good processability and low cost, epoxy resins are used as one of the most versatile thermosetting polymers with a wide range of applications including coatings, adhesives, structural composites and electronic materials [1,2,3]. However, their inherent brittle nature because of high degree of chemical crosslinking severely limits their uses in many applications. Up to now, various modifiers, such as rubber [4], thermoplastic [5], clay [6], hyperbranched polymers (HBPs) [7,8], nanomaterial [9,10] and layered double hydroxides (LDHs) [11] have been incorporated into epoxy resin, which can significantly improve the toughness of the epoxy resin. However, the key point for all these approaches is to toughen epoxy resin without sacrificing its strength, modulus and other thermal properties.

In recent years, the fast depletion of petroleum reserve and increasing environmental problems have led to a growing interest in the use of bio-based sustainable feedstock in the synthesis of bio-based chemicals and products. In this regard, some researches had focused on the synthesis and utilization of renewable material as efficient epoxy modifier [12,13,14,15,16]. Liu reported an approach to toughen epoxy resin with lignin, which could significantly toughen and simultaneously reinforce the epoxy resin. However, the glass transition temperature (*T*_g_) of the resin was reduced to a certain extent [13]. Satheesh reported the utilization of chitosan to modify epoxy resin and investigated the influence of chitosan loading on the thermal, mechanical, and morphological properties. The results showed that when the chitosan loading increased above 5 wt %, chitosan tended to agglomerate in epoxy resin, with the formation of clear phase separation. What is more, the tensile strength decreased after adding chitosan [14]. It is important to note that toughening an epoxy resin by bio-based modifiers without trade-offs in its modulus, mechanical strength, and thermal properties is still a big challenge.

Tannic acid (TA) is water-soluble high molecular weight polyphenolic compounds, mostly extracted from plants and microorganisms. It has a macromolecular structure composed of gallic units and abundant terminal phenolic hydroxyl groups. Owing to such a structure, TA shows remarkable properties and is widely used in many application, such as coatings, adsorption and antibacterial materials, mucoadhesive compounds, separator for lithium-ion batteries, and nanomaterials [17,18,19,20,21].

It has been proved that a highly branched structure can introduce more internal cavities or free volumes in cured thermosets, which is favor of improving toughness [22,23,24]. In addition, an aromatic structure is beneficial to the *T*_g_ and modulus of cured epoxy thermoset. In this context, the use of tannic acid as an epoxy modifier seems to be an interesting proposition because its architectural structure is similar to the hyperbranched aromatic polyester with abundant terminal phenolic hydroxyl groups. Furthermore, the utilization of bio-based tannic acid will contribute to the long-term sustainability including environmental and health safety issues [25].

Therefore, the motivation for this work is to utilize TA as a bio-based modifier for epoxy resins with simultaneous improvement in toughness and other properties. We had tried to add TA into epoxy formula without any modification. However, because of intermolecular hydrogen bonds, Van der Waals interactions and π–π stacking of aromatic groups, TA is immiscible with epoxy resin and tends to precipitation during curing. Therefore, a certain extent of chemical modification of TA is necessary to improve the miscibility of TA in epoxy matrix and enhance the interface interaction between TA and epoxy matrix.

In the study reported herein, a carboxylic acid-modified tannic acid (TA–COOH) was prepared through the simple esterification between TA and a commercial epoxy hardener methylhexahydrophthalic anhydride (MeHHPA). In addition, TA–COOH was added into the epoxy formula and used as an all-purpose epoxy modifier. It was found that TA–COOH could significantly improve the toughness of cured thermosets with an increase in impact strength under low loading amount, and simultaneously improve the elongation at break, *T*_g_ and strength. In addition, other thermal properties and fracture surfaces were also studied.

## 2. Experimental Section

### 2.1. Materials

**Scheme 1:**
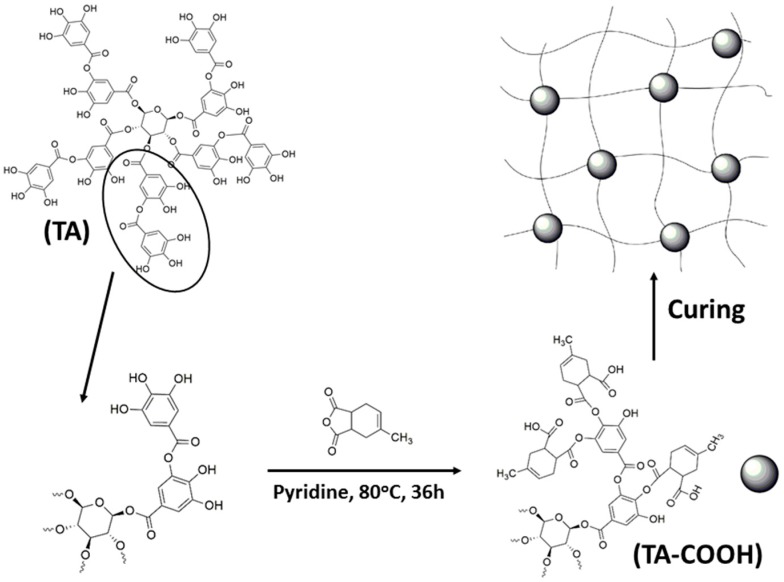
Synthetic route of TA–COOH.

TA (the main component is a kind of polygallic acid as given in Scheme 1, whose purity is 99%) and MeHHPA (99%) were purchased from Aladdin (Shanghai, China). Epoxy resin (diglycidyl ether of bisphenol A, trade name E-51) with an epoxy equivalent weight of 171–175 g per equivalent was obtained from Kukdo Chemical (Kunshan, China). All other reagents were of analytical grade and used as received from Sinopharm Chemical Reagent (Shanghai, China).

### 2.2. Synthesis of Carboxylic Acid-Functionalized Tannic Acid (TA–COOH)

TA (3.4 g, 2 mM) was dissolved in 30 mL of pyridine in a round-bottomed flask with a magnetic stirrer and a gas inlet to fill the flask with N_2_. Then 16.8 g of MeHHPA (100 mM) was added to the solution. The mixture was stirred at 80 °C for 36 h. After reaction, the mixture was precipitated into diethyl ether to remove pyridine and the unreacted MeHHPA. The precipitate was dissolved in THF and precipitated into diethyl ether twice, and then dried under vacuum at 30 °C to give a brown solid product.

### 2.3. Fabrication of TA–COOH/Epoxy Thermosets

The TA–COOH/epoxy thermosets were prepared by the following procedure. A required amount of TA–COOH was dispersed in epoxy resin (E-51) under mechanical stirring at 60 °C. Then, the curing agent (MeHHPA) and catalyst (ethyl triphenyl phosphonium bromide) were added to the above mixture (the epoxy and curing agent were in a 1:1 equivalent ratio, and the catalyst loading was 1 wt % of the total weight). Finally, the mixture was degassed and poured into mold. The samples for thermal and mechanical characterization were cured using the following profile: 80 °C for 1.5 h, 100 °C for 1 h, 120 °C for 1 h, and 140 °C for 2 h. These thermosets with 0.5, 1.0 and 2.0 wt % of TA–COOH were coded as TA–COOH0.5, TA–COOH1.0 and TA–COOH2.0, respectively. Neat epoxy was prepared following the same procedure as mentioned above.

### 2.4. Characterization

The ^13^C-NMR spectra of TA–COOH were recorded on a Bruker AV400M nuclear magnetic resonance spectrometer (400 MHz, Bruker, Karlsruhe, Germany). In order to calculate the degree of modification, an inverse gated decoupling technique with the time of 4 h was used. Fourier transform infrared (FTIR) spectra were obtained using a Thermo Fisher Scientific Nicolet iS50 spectrometer (Thermo Fisher Scientific, Waltham, MA, USA) at room temperature in the wavenumber range of 600–4000 cm^−1^. TGA experiment was carried out on a METTLER TOLEDO TGA/1 (Mettler Toledo, Greifensee, Switzerland) in N_2_ atmosphere with a heating rate of 10 °C min^−1^. DSC was recorded on a NETZSCH 204 F1 thermal analyzer (Mettler Toledo, Greifensee, Switzerland). The samples of ~5 mg in weight were placed in aluminum pans under nitrogen atmosphere. Dynamic mechanical analysis (DMA) was conducted on a TA Instruments DMA Q800 (TA Instruments, Newcastle, UK) at a heating rate of 3 °C min^−1^ and a frequency of 1 Hz under an air atmosphere. The linear coefficients of thermal expansion were measured using a METTLER TOLEDO TMA/SDTA841e thermal mechanical analyzer (Mettler Toledo, Greifensee, Switzerland). The tensile strengths of the cured hybrids were characterized by an Instron 1185 test machine (Instron Corp., Canton, MA, USA) according to ISO 527:1993. Un-notched impact strength tests were performed on a Ceast Resil impact tester (CEAST, Turin, Italy) according to ISO 179:1982. For each composition, at least 5 samples were measured. The fracture surfaces from fracture toughness tests were investigated by a HITACHI S-4800 field-emission scanning electron microscope (FESEM, HITACHI, Tokyo, Japan). Rheological measurements were performed on a discovery DHR-2 hybrid rheometer (TA Instruments, Newcastle, UK) equipped with cone and plate geometry (25 mm cone diameter, 1.986° cone angle, 50 mm gap size). All the experiments were performed at 25 °C.

## 3. Results and Discussion

### 3.1. Synthesis and Characterization

**Figure 1:**
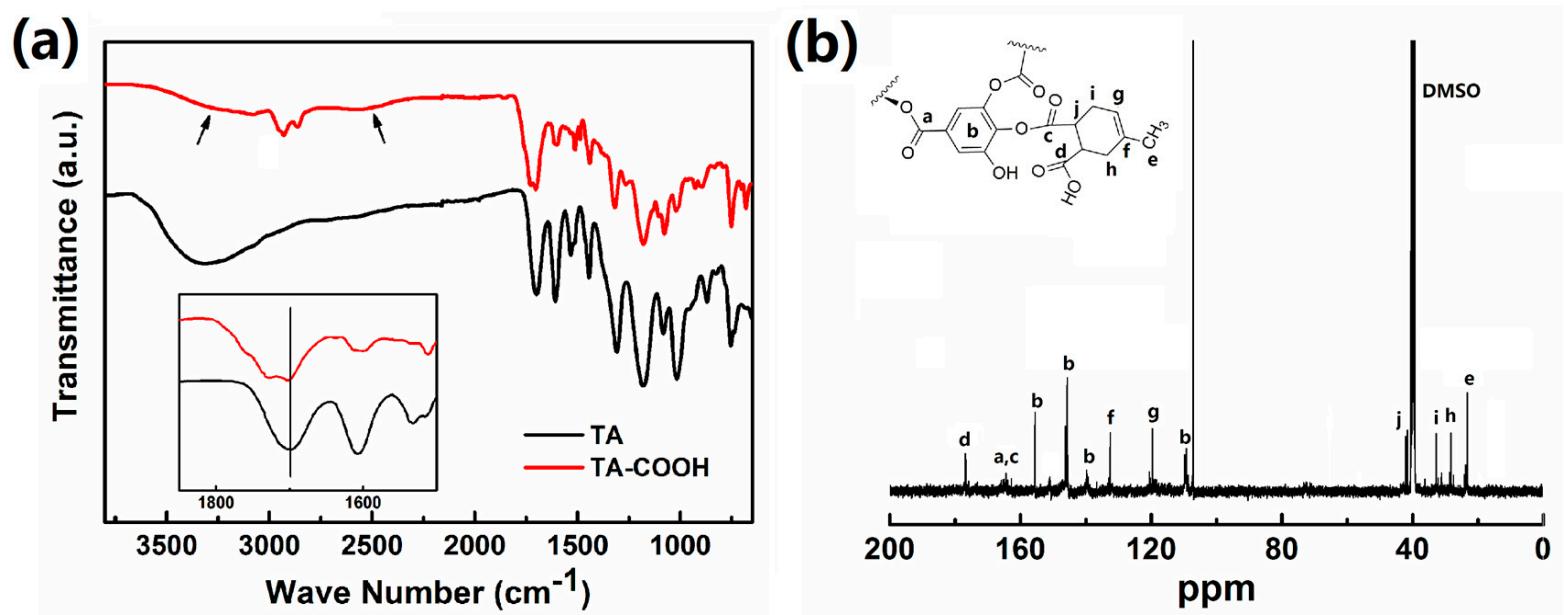
(**a**) FTIR spectra of TA and TA–COOH and (**b**) ^13^C-NMR spectrum of TA–COOH.

The commercial TA is given as C_76_H_52_O_46_. Its chemical structure was given in Scheme 1. It has 10 esterified galloyl groups linked to a glucose core. Theoretically, it contains 25 phenolic hydroxyl groups, which could serve as reactive sites and be exploited for the functionalization of TA through various reactions [26,27,28]. In this work, TA was modified by a pyridine-catalyzed esterification with a common epoxy hardener MeHHPA, generating terminal carboxyl groups. The terminal carboxyl groups could react with epoxide groups in curing process and then provide a good interface interaction between TA–COOH and epoxy matrix. The proposed structure of TA–COOH is shown in Scheme 1. First, the characterization of the TA–COOH was carried out by FTIR spectroscopy, as shown in Figure 1a. TA shows a broader OH band, which is attributed to the great hydrogen bonding interaction of phenol groups. After modification, the OH region weakens and a new carboxylic group band in the region of 2500−3500 cm^−1^ appears (as arrow has pointed out). The carbonylic region was also given in the inset. As can be seen, compared to the IR spectrum of TA, a new absorption peak at 1730 cm^−1^ occurrs, which could be assigned to the stretching vibration of C=O in the ester groups generated by the ring-opening of anhydride. Moreover, the bands at 2804 cm^−1^ and 2959 cm^−1^ are due to the aliphatic C–H vibrations of MeHHPA moiety in the TA–COOH. 

The structure of TA–COOH was further confirmed by ^13^C-NMR spectroscopy. Figure 1b shows the ^13^C -NMR spectrum of TA–COOH with the corresponding assignments. It can be seen, after modification, TA–COOH shows a resonance at 176 ppm, which can be assigned to the –COOH groups generated by anhydride. The resonances at 23, 29, 33, 41, 119 and 133 ppm are assigned to the aliphatic C from MeHHPA moiety. The resonances at 109, 140, 146, 151 and 155 ppm are assigned to the C in benzene ring from TA. 

Because of steric hindrance affect, not all the 25 hydroxyl groups of TA are equally active. Thus, the degree of modification of MeHHPA was calculated from the integration of the aliphatic and aromatic signals. After calculation, it is found that about 15 hydroxyl groups of TA reacted with MeHHPA. Although the modification degree is not as high as expected, TA–COOH still shows good solubility in epoxy resin by stirring at 60 °C for 2 h. In addition, the terminal carboxyl groups offer a good interface interaction between TA–COOH and epoxy matrix.

### 3.2. Rheological Properties

**Figure 2:**
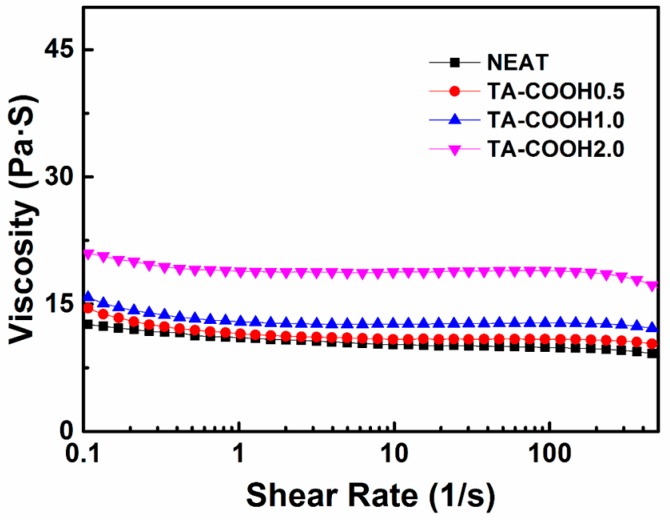
Viscosity versus shear rate of neat epoxy and TA–COOH modified epoxy resins.

Generally, when epoxy resin is toughened by a linear liquid rubber, its viscosity increases to a great extent. However, for hyperbranched toughener, it usually has a low melt viscosity because of its globular structure. Such a low melt viscosity is good for processing, which shows a very little increase in the viscosity than liquid rubber. The rheological studies of the neat epoxy and epoxy resin with different TA–COOH loading were performed. All of the samples have Newtonian behaviors and show similar rheological behavior in the range of frequencies studied (Figure 2). As expected, the viscosity values of the samples with different TA–COOH loadings show only a little increase as that of neat epoxy. Such a low viscosity is still benefit for processability.

### 3.3. Curing Study

**Figure 3:**
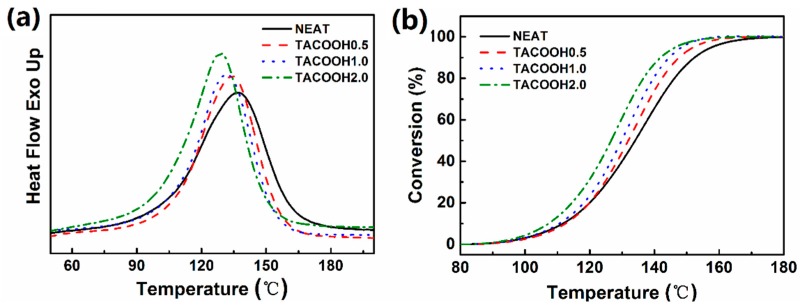
(**a**) DSC thermograms of neat epoxy and the TA–COOH modified epoxy resins and (**b**) degree of conversion against temperature of the curing of neat epoxy and the TA–COOH modified epoxy resins.

As an aim to maintain a certain interface interaction between the TA–COOH and the epoxy matrix after curing, we selected anhydride as curing agent. The use of anhydride as curing agents has been extensively reported [29,30]. Here the influence of introducing TA–COOH on the curing behavior of epoxy/anhydride system was studied. Figure 3 compares the DSC and conversion curves of the neat epoxy and the samples with different TA–COOH loadings. As is mentioned above, the use of TA–COOH produces an increase in the viscosity of the mixture. Thus, it would decrease the mobility of the propagating species and lower the reaction activity. However, compared to the neat epoxy system, the TA–COOH modified epoxy resin systems show lower curing temperature and the curing temperature decreases with increase the loading of TA–COOH. This implied that the incorporation of TA–COOH could accelerate the epoxy/anhydride curing reaction.

The curing kinetics were studied by the non-isothermal integral isoconversional procedure. The activation energy (*E*_a_) and pre-exponential factor (*A*) was calculated and the results were shown in Table 1. As we can see, the pre-exponential factor decrease slightly, however, the activation energy also decreases to a certain extent. In consideration of the compensation effect between activation energy and pre-exponential factor, we consider the curing process was accelerated by TA–COOH. As we said above, the viscosity did not increase a lot, such an acceleration affect could be explained as follows: After the modification of TA with MeHHPA, TA–COOH contains terminal carboxyl groups and some unreacted hydroxyl groups. The carboxyl groups can serve as epoxy resin hardener. In the meantime, the carboxyl groups and residual hydroxyl groups can initiate the polycondensation mechanism of reaction between epoxide and anhydride, accelerating the curing [31,32].

### 3.4. Dynamic Mechanical Properties

**Figure 4:**
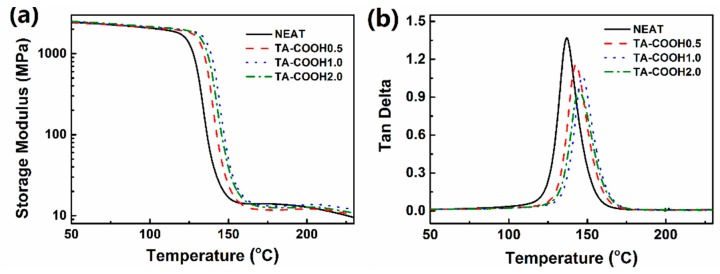
Storage modulus (**a**) and tan delta (**b**) versus temperature for the neat epoxy and TA–COOH modified epoxy resins.

The dynamic mechanical behaviors of neat epoxy and the TA–COOH modified epoxy resins were measured. The storage moduli (*E*’) and loss tangent (tan δ) as a function of temperature for the cured neat epoxy and the thermosets containing 0.5, 1.0 and 2.0 wt % TA–COOH are shown in Figure 4, and the data are summarized in Table 1. Compared with neat epoxy, the TA–COOH modified epoxy shows a slightly decrease in the rubbery plateau modulus (*E*_r_). Following classical rubber elasticity, *E*_r_ is proportional to the average crosslinking density. The crosslinking density (ρ) of a cured epoxy network can be calculated using the equation:

(1) $$\rho =\frac{E_{r}}{3RT}$$

Where ρ represents the crosslinking density per unit volume (mol·cm^−3^), *E*_r_ is rubbery modulus (MPa), *R* is the gas constant, and *T* is the absolute temperature. Theoretically, adding of TA–COOH may enhance the crosslinking density due to its higher functionality. However, the thermosets with different TA–COOH loadings show lower crosslinking density than neat epoxy. This presumably can be explained as following. Although the carboxylic content of TA–COOH is relative low, which has been confirmed by ^13^C-NMR (Figure 1), the external phenolic hydroxyl groups can also react with epoxy resin in some ways. This makes the TA–COOH an extra hardener. As the ratio of epoxy resin and hardener kept the same in all the formulas, the adding of TA–COOH would lead to an unbalanced formulation, which may decrease the crosslinking density. On the other hand, the incorporation of bulk TA–COOH unit into the cured network enlarged the average molecular weight between crosslinks, thus lead to a decrease in crosslinking density. With such a complex mechanism, the thermoset with 1.0 wt % TA–COOH loading shows the maximum of crosslinking density. Similar maxima at 1.0 wt % loadings are also found in *T*_g_ and linear coefficients of thermal expansion (see later sections).

Tan δ is defined as the ratio of the loss modulus to the storage modulus, and the peak of the tan δ versus temperature curve is taken as *T*_g_. For all of the thermosets, the curves are unimodal and only one *T*_g_ is observed. That is, in all cases the network structures are homogeneous and no obvious phase separation occurs. Below a loading of 1.0 wt %, *T*_g_ increases with TA–COOH loading and reaches a highest value of 146.7 °C, which is 10 °C higher than that of neat epoxy. For the thermoset with 2.0 wt % TA–COOH loading, *T*_g_ slightly decreases. *T*_g_ was also measured by DSC and the results were consistent with the results of DMA. Generally, decreasing the crosslink density alone would decrease the material rigidity, thereby reducing *T*_g_. However, *T*_g_ is affected by both the crosslinking density and the chain flexibility. As is mentioned, the crosslinking density decreases after adding TA–COOH. Therefore, the increase of *T*_g_ is probably due to the high content of aromatic structure in TA–COOH, which partly enhances the chain rigidity.

### 3.5. Thermal Expansion

The values of the coefficient of thermal expansion (CTE) measured in the glassy region (α_g_) and the rubber region (α_r_) as well as their difference (Δα = α_r_ − α_g_) are listed in Table 2. Compared with neat epoxy, all the TA–COOH modified epoxy resins show lower CTE values in the glassy region. Such a small α_g_ in cured hybrids could lower the internal stress when processing and is very beneficial for the composite matrix, because it helps to maintain better interfacial strength during temperature cycles and shocks. On the other hand, as shown in Table 2, when the TA–COOH loading increases, α_g_ decreases, and *α*_r_ increases. Thus, the Δα increases. Based on the free volume theory [33], the fractional free volume at temperature *T* (*f*_T_) can be expressed as:

(3) $$f_{T}\;=\;f_{g}\;+\Delta \alpha \left(T\;-\;T_{g}\right)$$

Where Δα = α_r_ − α_g_ is the difference between the CTE in rubbery and glassy states, and *f*_g_ is the fractional free volume at *T*_g_. Therefore, the increased Δα for the TA–COOH modified epoxy resins indicates the existence of more free volume when TA–COOH is incorporated into the epoxy networks. Such a result is also observed in other reports that introducing a hyperbranched polymer into epoxy networks can increase the free volume. It should be noted that these increase in free volume could significantly improve the toughness in cured thermosets [22,23,24,34,35,36].

### 3.6. Mechanical Properties

**Figure 5:**
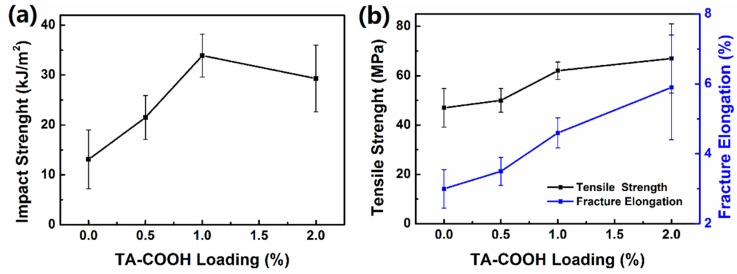
Impact (**a**) and tensile (**b**) properties of the neat epoxy and TA–COOH modified epoxy resins with different TA–COOH loadings.

To investigate the reinforcing and toughening effects of the incorporated TA–COOH, the thermosets with different TA–COOH loadings were prepared and their tensile and impact properties were tested. From Figure 5a, it can be found that the impact strength was distinctly improved with the incorporation of TA–COOH. It reaches the maximum of 33.9 kJ/m^2^ at 1.0 wt % loading, which is 159% higher than that of neat epoxy. The further increase of TA–COOH loading over 1.0 wt % leads to a slightly decrease in the impact strength, which can be ascribed to the “crosslinking density reduction” effect.

The tensile test results are shown in Figure 5b. The elongation at break increases continuously from about 3.0% to 5.9% with increasing TA–COOH loading from 0 to 2.0 wt %. Such an increase should be related to the good interface interaction via a chemical reaction between the terminal carboxyl group of TA–COOH and epoxy matrix. For typical epoxy material, enhancing the elongation at break usually accompanies with decreasing tensile strength. However, a continuous increase in tensile strength was also observed as the loading of TA–COOH increases. The thermoset with 2.0 wt % TA–COOH loading has a maximum tensile strength of 67 MPa, which is about 42.5% higher than that of neat epoxy. The increase in tensile strength may be due to the aromatic structure and good interface interaction between TA–COOH and epoxy matrix.

### 3.7. Morphology of Fractured Surfaces

**Figure 6:**
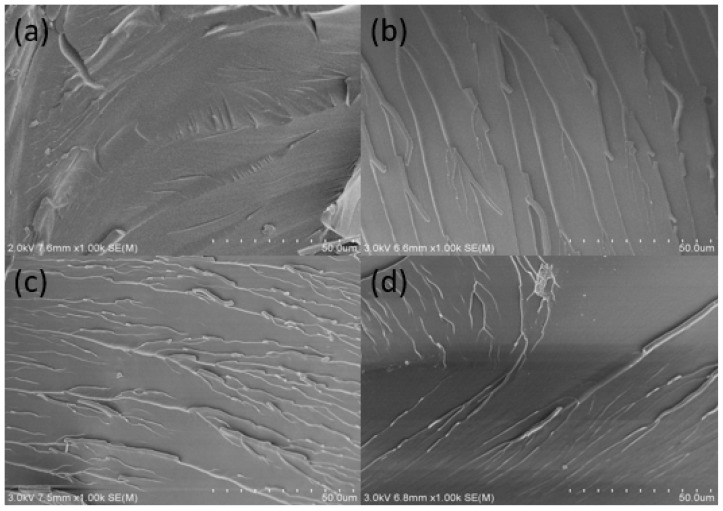
SEM images of the impact fracture surfaces of (**a**) neat epoxy; (**b**) TA–COOH0.5; (**c**) TA–COOH1.5; and (**d**) TA–COOH2.0.

The morphology of the fracture surfaces of neat epoxy and the TA–COOH modified epoxy resins were investigated by SEM. Figure 6 presents the SEM micrographs of impact fractured surfaces of the thermosets prepared. It can be observed that the fracture surface of neat epoxy (Figure 6a) is very smooth except for some river-like lines, indicating a brittle failure mode without any ductility. In contrast, the fracture surfaces of the thermosets with TA–COOH are much rougher than that of the neat epoxy and without traces of phase separation. In addition, a lot of oriented “protonema” or “fibrils” are clearly observed, which indicates that the thermosets undergo more plastic deformation. As we already know, large plastic deformation and crazing processes could significantly absorb the energy, thus result in an increase in the amount of energy needed for crack propagation and for the formation of new surfaces. Such a result was also observed by other researchers and this is in good agreement with in situ toughening mechanism [36,37,38,39].

### 3.8. Thermogravimetric Analysis

**Figure 7:**
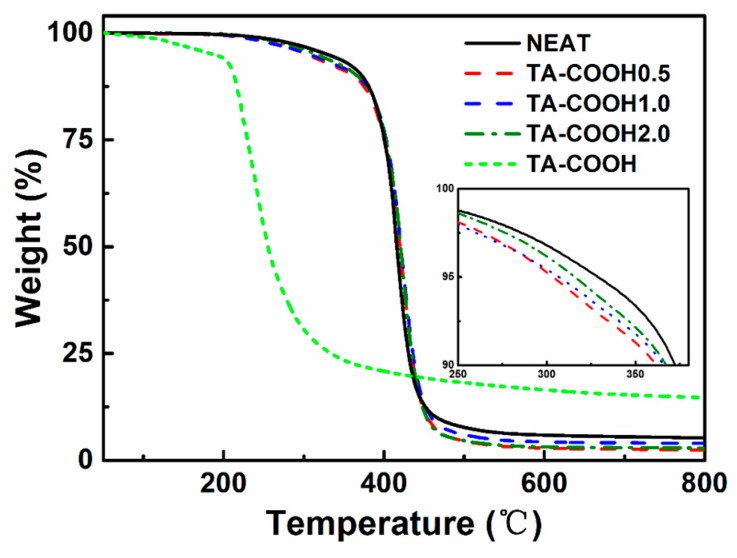
TGA curves of the neat epoxy and TA–COOH modified epoxy resins.

TGA curves of cured neat epoxy and thermosets with different TA–COOH loadings are shown in Figure 7. As we can see, both neat epoxy and TA–COOH modified epoxy show only one similar degradation stage, indicating that a homogenous structure of the matrix is formed and the breakage of bonds in the network structure occurs simultaneously. After careful observation, it can be seen that the initial degradation temperatures (*T*_5%_) of the thermosets with different TA–COOH loadings are slightly lower than that of neat epoxy (as shown in the inset of Figure 7). In addition, it is worth mentioning that the *T*_5%_ increases with an increase in the amount of TA–COOH. The combination of some effects could explain this experimental behavior. On the one hand, the thermal stability of TA–COOH is relatively low, its *T*_5%_ was about 200 °C. On the other hand, the terminal carboxyl groups of the TA–COOH provide miscibility during curing and allow reaction with other network functional groups, which in turn produces greater adhesion to the matrix and may prevent the elimination of volatile fragments.

## 4. Conclusions

A bio-based carboxyl-terminated tannic acid (TA–COOH) had been synthesized through a simple esterification between TA and MeHHPA. Then TA–COOH was used as a modifier for anhydride cured epoxy system. Owing to the chemical modification, TA–COOH could easily disperse in epoxy resin and showed a good interface interaction between TA–COOH and epoxy matrix. When TA–COOH was used as modifier, it can simultaneously improve toughness, elongation at break, *T*_g_, and strength. Especially for the thermoset with 1.0 wt % TA–COOH loading, it showed the impact strength and tensile strength of 33.9 kJ/m^2^ and 62.0 MPa, respectively, which are 159% and 32% higher than that of neat epoxy, respectively. In the meantime, the *T*_g_ increased to 146.7 °C. According to the results of DMA, TMA and SEM, no phase separation occurred. The simultaneous enhancements in *T*_g_, tensile strength, and impact strength are mainly because of the synergistic effect of aromatic structure, decreasing of cross linking density, increasing of free volume and good interface interaction.

## Figures and Tables

**Table 1:** DMA and DSC results of the neat epoxy and TA–COOH modified epoxy resins.

Sample	*E*_a_^a^ (kJ/mol)	ln *A* (min^−1^)	*T*_g_ (°C)	*E*_g_^b^ (MPa)	*E*_r_^c^ (MPa)	ρ (10^−3^ mol/cm^3^)
NEAT	68.3	9.15	136.8	2,515	13.9	1.27
TA–COOH0.5	-	-	142.6	2,412	11.7	1.05
TA–COOH1.0	63.5	7.78	146.7	2,421	13.2	1.18
TA–COOH2.0	-	-	144.6	2,500	12.7	1.14

^a^ Apparent activation energy at 50% conversion; ^b^ Storage modulus at 50 °C; ^c^ Storage modulus at *T*_g_ + 30 °C.

**Table 2:** Linear coefficients of thermal expansion determined from TMA measurements.

Sample	α_g_	α_r_	Δα = α_r_ − α_g_
	(× 10^−6^ K^−1^)	(× 10^−6^ K^−1^)	(× 10^−6^ K^−1^)
NEAT	81.3	180.1	98.8
TA–COOH0.5	76.4	190.6	114.2
TA–COOH1.0	67.9	198.7	130.9
TA–COOH2.0	75.5	200.1	124.6
